# Palaeoecological records of coral community development on a turbid, nearshore reef complex: baselines for assessing ecological change

**DOI:** 10.1007/s00338-017-1561-1

**Published:** 2017-03-04

**Authors:** J. A. Johnson, C. T. Perry, S. G. Smithers, K. M. Morgan, N. Santodomingo, K. G. Johnson

**Affiliations:** 1 School of Geography, College of Life and Environmental Sciences, University of Exeter, Exeter, EX4 4RJ UK; 2 College of Marine and Environmental Sciences, James Cook University, Townsville, QLD 4811 Australia; 3 Department of Palaeontology, Natural History Museum, Cromwell Road, London, SW7 5BD UK

**Keywords:** Ecological baselines, European settlement, Great Barrier Reef, Inner shelf, Palaeoecology, Water quality

## Abstract

Understanding past coral community development and reef growth is crucial for placing contemporary ecological and environmental change within appropriate reef-building timescales. On Australia’s Great Barrier Reef (GBR), coral reefs situated within coastal inner-shelf zones are a particular priority. This is due to their close proximity to river point sources, and therefore susceptibility to reduced water quality discharged from coastal catchments, many of which have been modified following European settlement (ca. 1850 AD). However, the extent of water-quality decline and its impacts on the GBR’s inner-shelf reefs remain contentious. In this study, palaeoecological coral assemblage records were developed for five proximal coral reefs situated within a nearshore turbid-zone reef complex on the central GBR. A total of 29 genera of Scleractinia were identified from the palaeoecological inventory of the reef complex, with key contributions to reef-building made by *Acropora*, *Montipora*, and *Turbinaria.* Discrete intervals pre- and post-dating European settlement, but associated with equivalent water depths, were identified using Bayesian age–depth modelling, enabling investigation of competing ideas of the main drivers of nearshore coral assemblage change. Specifically, we tested the hypotheses that changes in the composition of nearshore coral assemblages are: (1) intrinsically driven and linked to vertical reef development towards sea level, and (2) the result of changes in water quality associated with coastal river catchment modification. Our records found no discernible evidence of change in the generic composition of coral assemblages relative to European settlement. Instead, two distinctive depth-stratified assemblages were identified. This study demonstrates the robust nature of nearshore coral communities under reported water-quality decline and provides a useful context for the monitoring and assessment of ecological change on reefs located within the most nearshore turbid-zone environments of the central GBR.

**Electronic supplementary material:**

The online version of this article (doi:10.1007/s00338-017-1561-1) contains supplementary material, which is available to authorized users.

## Introduction

Nearshore coastal environments that experience high levels of sedimentation and turbidity are widely perceived as ‘marginal’ (sensu Perry and Larcombe 2003) for coral growth and reef development. This is because sediments, especially fine-grained terrigenous sediments, can reduce coral fecundity and survival (Erftemeijer et al. 2012; Jones et al. 2015). Coral reefs located in environments exposed to regimes of naturally high sediment flux (defined by the total mass of sediment deposited and resuspended; Browne et al. 2012c) are often referred to as ‘turbid-zone reefs’. On Australia’s Great Barrier Reef (GBR), turbid-zone reefs occur across a range of geomorphic settings, located within a shallow (<20 m deep) inner-shelf zone which extends up to 20 km offshore of the mainland coast (Larcombe and Woolfe 1999b; Browne et al. 2012a). The inner shelf of the GBR is characterised by the presence of fine-grained terrigenous sediments which form an inshore sediment prism that extends offshore towards a seaward limit close to the 15-m isobath (Woolfe and Larcombe 1999). In the shallow (<5 m depth) coastal areas of the inner shelf, these fine-grained sediments are frequently resuspended by waves, producing wide fluctuations in turbidity (Larcombe et al. 2001; Browne et al. 2012b). Based on the prevailing environmental conditions, a distinction has recently been made between inner-shelf reefs that occur within coastal ‘nearshore’ (<10 m isobath) areas and those located further offshore in more ‘inshore’ settings (>10 m isobath), where the local natural sedimentary background conditions are less extreme (Morgan et al. 2016a).

Despite the sedimentary setting of the GBR inner shelf, the reefs located within this zone can support diverse coral communities. Regional species richness typically exceeds 60 species across the entire inner shelf (Sweatman et al. 2007) and live coral cover averages ~20% (www.aims.gov.au/docs/data-centre/reef-monitoring-surveys.html), but can be as high as ~40% (e.g. Browne et al. 2010; Morgan et al. 2016a). However, declining water quality is considered a major threat to the health of inner-shelf reefs across the GBR (Brodie et al. 2012). Since European settlement (ca. 1850 AD), the annual input of sediment into the GBR lagoon is estimated to have increased by a factor of 5.5, while inputs of nitrogen and phosphorus are estimated to have increased by approximately six- and ninefold, respectively (Lewis et al. 2007; Kroon et al. 2012). In spite of these reported increases in sediment and nutrient loads, the impact of declining water quality on the GBR’s inner-shelf reefs remains contested. Central to the debate is uncertainty related to the impact of increased sediment loads relative to the natural movement and resuspension of sediments which have accumulated on, and characterised, the inner shelf over the last ~6000 yr (Larcombe and Woolfe 1999b).

Divergent ecological trajectories further confound the issue, with some studies reporting long-term coral community stability under the continuous influence of terrigenous sediments (Perry et al. 2008, 2009; Roche et al. 2011; Ryan et al. 2016), while others have linked shifts in coral assemblages to anthropogenically induced water-quality decline (e.g. Fabricius et al. 2005; Roff et al. 2013). One explanation for the apparent disparity between existing ecological records is that the impact of increasing sediment influx is spatially heterogeneous, with an emerging hypothesis that nearshore coral communities are pre-adapted to, and thus better able to cope with, conditions of low light availability and sedimentation (Perry et al. 2008; Morgan et al. 2016a; Ryan et al. 2016). This pre-adaption may therefore have instilled a greater resilience within these nearshore coral communities to changes in water quality than those of inshore reefs, which are located further offshore towards the inner/mid-shelf boundary, and where the influence of suspended sediments and the frequency of exposure to river flood plume-associated particulates (i.e. nutrients and solids) is lower (Devlin and Brodie 2005; Perry et al. 2008; Morgan et al. 2016a).

On the GBR, palaeoecological investigations have provided valuable insights into the regional controls and long-term reef development of the most nearshore coral communities (see Browne et al. 2012a for review). An important concept to emerge from these studies is that changes in nearshore coral assemblages are intrinsically driven and related to shallowing environmental conditions during vertical reef development towards sea level (e.g. Perry et al. 2008, 2009; Roche et al. 2011; Ryan et al. 2016). To date, however, studies of nearshore coral assemblage changes, over appropriate reef-building timescales (i.e. >decadal), have largely been restricted to individual reefs that have attained sea level (i.e. reefs within the late stages of ‘geomorphological maturity’ sensu Hopley 1982). Here, we use a core-based approach to develop detailed palaeoecological coral assemblage records for five proximal nearshore reefs presently existing at different stages of geomorphological development. Established palaeoecological records were used to test competing ideas of the main drivers of nearshore coral assemblage change within the central GBR. Specifically, we test the hypotheses that changes in the composition of nearshore coral assemblages are: (1) intrinsically driven and linked to vertical reef development towards sea level, and (2) the result of changes in water quality associated with coastal river catchment modification following European settlement.

## Materials and methods

### Study site

**Fig. 1:**
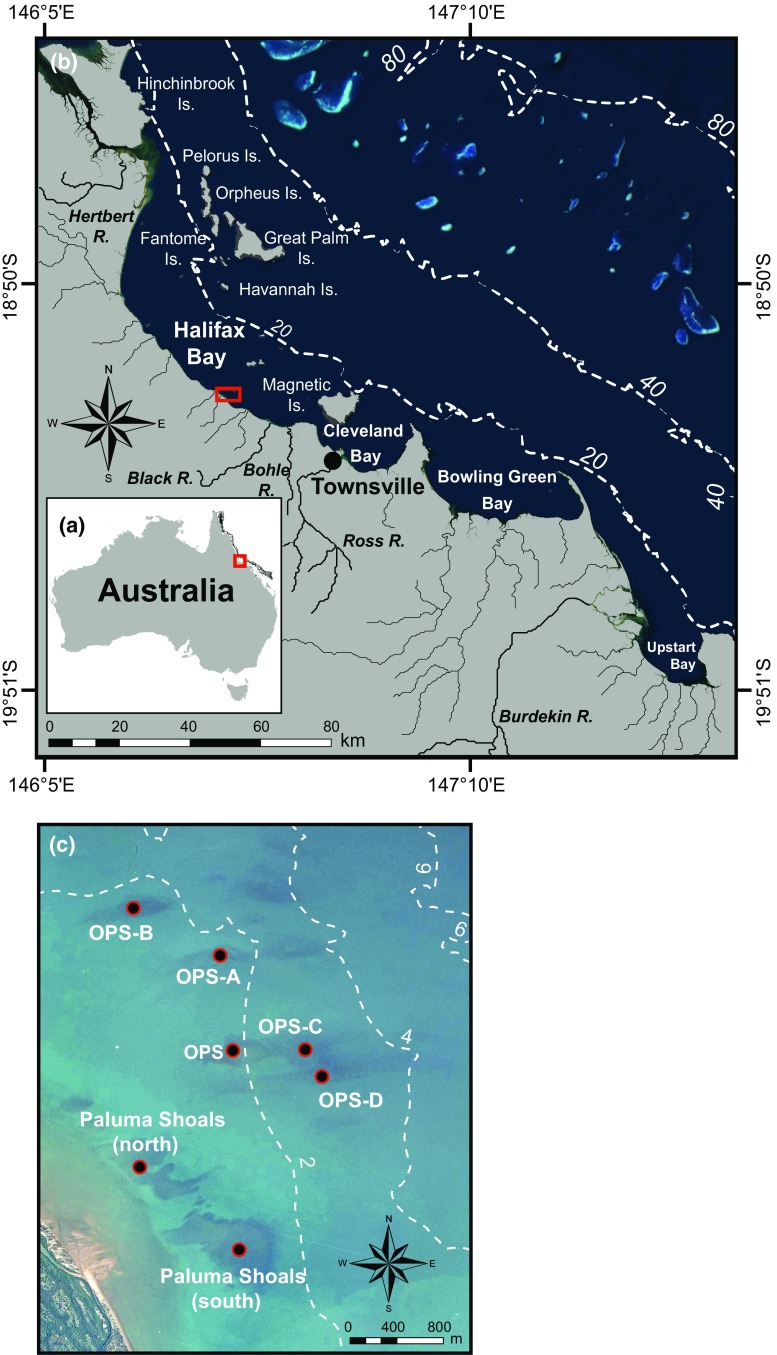
Site maps showing the location of **a** Halifax Bay, Queensland, Australia; **b** the Paluma Shoals Reef Complex (PSRC) in Halifax Bay, central Great Barrier Reef (*red box*); and **c** an aerial image of the PSRC and its constituent reefs: Paluma Shoals (north and south shoal), Offshore Paluma Shoals (OPS), Offshore Paluma Shoals A, B, C and D (OPS-A, B, C and D). The presented isobath contours are in metres and are derived from Beaman (2010)

The Paluma Shoals reef complex (PSRC) comprises seven nearshore coral reefs distributed across an area of ~16 km^2^, located in Halifax Bay on the central GBR (Fig. 1; Morgan et al. 2016a). With the exception of Paluma Shoals (north shoal), the reefs of the PSRC are detached from the mainland and extend up to 3 km offshore. Presently, the reefs of the PSRC are at various stages of geomorphological development (see Morgan et al. 2016a, b; Fig. 1) ranging from: (1) the fully submerged reef structures of Offshore Paluma Shoals A, B, C, and D (OPS-A, B, C, and D); (2) the reef structure of Offshore Paluma Shoals (OPS) that is emergent at lowest astronomical tide (LAT); and (3) Paluma Shoals (north and south shoal) that have attained sea level.

Halifax Bay is a shallow (<20 m water depth), low-gradient (<1:1000) embayment, with a semi-diurnal tidal cycle and a spring tide maximum of 3.8 m (Larcombe et al. 2001). In the PSRC, water turbidity is strongly influenced by wave activity, with suspended sediment concentrations increasing (>200 mg L^−1^) following exposure to larger waves (H_s_ > 0.5 m) generated by persistent winds (Browne et al. 2012b). On Paluma Shoals, average sedimentation rates vary among geomorphological zones, ranging from 0.9 g m^−2^ d^−1^ on the reef flat to 120 g m^−2^ d^−1^ in more sheltered leeward locations (Browne et al. 2012c).

### Core recovery

**Fig. 2:**
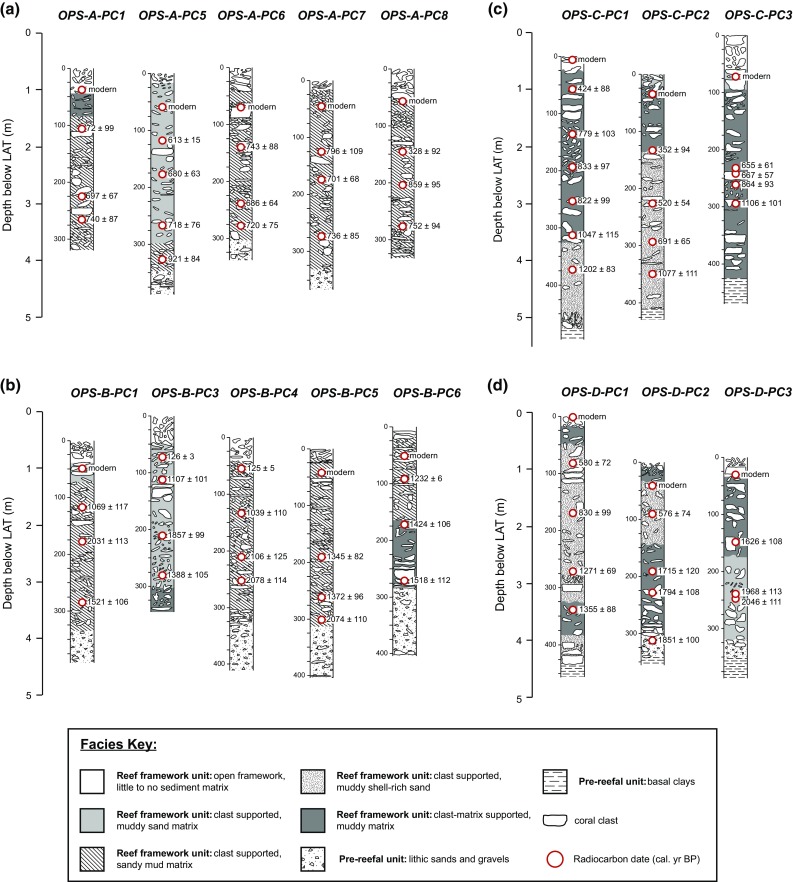
Stratigraphic diagrams and chronostratigraphies of the cores recovered from **a** Offshore Paluma Shoals (OPS)-A; **b** OPS-B; **c** OPS-C; and **d** OPS-D. All core depths (cm) have been uncompacted and are presented relative to lowest astronomical tide. The median probability ages of the available radiocarbon dates (Morgan et al. 2016b) are presented in calibrated years before present (cal. yr BP ± 1σ). The term ‘modern’ denotes ages associated with dates post-dating 1950 AD (0 cal. yr BP)

To reconstruct palaeoecological coral assemblages from the PSRC, 16 percussion cores (method described in Smithers and Larcombe 2003) were recovered across four separate reefs (OPS-A, B, C and D; Fig. 1) during the 2013 and 2014 winter (July), spring low-tide windows, using 95-mm-internal-diameter aluminium piping (Fig. 2). Core records were augmented by an additional five cores previously recovered from OPS in July 2010 (see Perry et al. 2013) and which have not previously been used for palaeoecological analysis. Water depth and time of measurement were recorded at each core location, allowing core elevations to be reduced to LAT. Core recovery was 100% in all cases, with an average penetration of approximately 4 m (Table 1). Importantly, all but four cores terminated in pre-reefal sedimentary sequences of lithic sands or stiff mottled clays (Fig. 2). Core compaction, calculated (assuming uniform compaction) using core length and penetration measurements, was variable and ranged from 21 to 52% (Table 1). Following collection, cores were split, photographed and logged (Fig. 2).

**Table 1:** Details of percussion cores collected from the Paluma Shoals Reef Complex

Reef	Core	Depth relative to LAT (m)	Core length (m)	Penetration (m)	Compaction (%)
OPS-A	PC-1	−0.63	2.2	3.0	32
	PC-5	−0.75	2.9	3.8	25
	PC-6	−0.70	2.6	3.8	32
	PC-7	−0.86	2.5	3.8	32
	PC-8	−0.66	1.9	3.4	43
OPS-B	PC-1	−0.52	2.6	4.0	35
	PC-3	−0.90	1.9	3.3	42
	PC-4	−0.45	2.4	4.0	41
	PC-5	−0.66	2.7	3.9	33
	PC-6	−0.29	2.8	4.1	33
OPS-C	PC-1	−0.41	2.8	5.0	44
	PC-2	−0.73	2.8	4.1	34
	PC-3	−0.50	2.6	4.6	45
OPS-D	PC-1	−0.70	2.5	4.5	44
	PC-2	−0.88	2.5	3.5	28
	PC-3	−0.80	1.8	3.8	52
OPS	PC-1	−0.25	3.5	4.5	21
	PC-2	−0.18	3.0	4.0	24
	PC-3	0.15	3.5	5.0	28
	PC-4	0.35	3.0	4.1	32
	PC-5	−0.15	2.6	3.7	28

### Taxonomic analysis

To determine the abundance of coral taxa, and to enable comparison of coral assemblages at equivalent water depths, all cores were divided into continuous 40-cm (uncompacted) intervals, relative to LAT. The interval size was selected as a compromise to be large enough to be ecologically meaningful (i.e. to adequately represent coral life assemblages), but small enough to obtain a sufficient number of samples from each core. Initially, each sample interval was wet-sieved to separate the fine-grained (<0.063 mm) and sand-sized (<2 mm) sediments from the coarser clastic materials. The retained materials (>2 mm) were then oven-dried before sieving (sieve aperture: 10 mm) to isolate individual coral clasts. For identification, collected clasts were grouped according to visible morphological traits, including: (1) overall growth morphology; (2) corallite arrangement and budding; (3) corallite morphology; and (4) coenosteum and internal corallite structure.

Over 18,000 individual coral clasts (~85 kg of coral material) were recovered and identified from the 21 cores. To ensure taxonomic consistency within this study, and to enable reliable comparisons to be made with previous investigations, corals were identified to genus. In order to build a palaeoecological species inventory for the PSRC, more detailed species-level taxonomic analyses were conducted on collected type specimens. Specimen identification followed Veron and Pichon (1976, 1980, 1984), and Cairns and Parker (1992), while corals of the genus *Acropora* followed descriptions by Wallace (1999). Species names and their spatial distributions were validated using online databases provided by the World Registry of Marine Species (WoRMS; www.marinespecies.org/) and the Ocean Biogeographic Information System (OBIS; www.iobis.org).

Overall, coral materials exhibited a high degree of preservation. Approximately 10% of the examined coral clasts were indeterminate and classified as ‘unidentified’. Generic abundances were determined by the relative contribution of each taxon to the total mass of identified coral within each sample interval. This method provides a good representation of fossil coral assemblage composition as it reduces the influence of preferential preservation which biases frequency-based abundance estimates towards corals with more fragile growth forms (i.e. branching and platy corals; Edinger et al. 2001; Santodomingo et al. 2016).

### Statistical analysis

#### Taxonomic richness

To assess the extent to which the sampling strategy captured the taxonomic richness of each reef, sample-based rarefaction analysis was conducted in EstimateS (version 9.1; Colwell 2013). The analysis followed methods described in Colwell et al. (2012), enabling the production of both rarefaction and extrapolation curves, together with their associated confidence intervals. To produce smooth curves, the analysis followed recommendations in Colwell (2013). Individual cores were treated as samples and randomised (resampled) 100 times, while curve extrapolations were conducted to twice the sample reference number.

#### Coral assemblages

Prior to further statistical analyses, ‘rare’ taxa (those accounting for <0.5% of the total mass of identified coral material) were excluded, following recommendations in Clarke and Warwick (2001) and based on the assertion that their abundance, as represented within fossil records, can be unreliable for statistical comparison (Bonelli et al. 2006). The identified ‘rare’ taxa included 13 genera, equating to approximately 400 clasts. Cumulatively, the ‘rare’ taxa accounted for less than 3% of the total mass of examined coral materials (Table 2). Only depth intervals with more than two core replicates per reef were included in statistical tests. All abundance data (per cent mass abundance) were fourth-root-transformed to reduce data asymmetry (Legendre and Birks 2012) unless stated otherwise.

**Table 2:** Relative contributions of coral genera to the total mass of identified coral material

Genus	Contribution (%)
*Acropora*	45.7
*Montipora*	22.5
*Turbinaria*	11.2
*Euphyllia*	4.4
*Porites*	2.8
*Favites*	2.2
*Cyphastrea*	1.5
*Galaxea*	1.4
*Goniopora*	1.3
*Hydnophora*	1.0
*Fungia*	0.9
*Pavona*	0.8
*Platygyra*	0.8
*Psammocora*	0.7
*Pachyseris*	0.6
*Alveopora*	0.5
Dipsastraea*	0.4
Oulophyllia*	0.4
Stylophora*	0.4
Duncanopsammia*	0.2
Lobophyllia*	0.2
Coscinarea*	0.1
Echinophyllia*	0.1
Leptoseris*	0.1
Oxypora*	0.1
Balanophyllia*	0.0
Echinopora*	0.0
Heterocyathus*	0.0
Pocillopora*	0.0

Specimen reference plates are available in ESM Figs. S1–S6

* Denotes genera that were identified as ‘rare’ (<0.5% contribution to the total mass of identified coral)

A two-way permutational multivariate analysis of variance (PERMANOVA; Anderson 2001) with 9999 permutations was used to characterise the coral assemblages preserved within the palaeoecological record of the PSRC and to investigate variations in assemblage composition among individual reefs and water depth intervals. Pairwise PERMANOVAs with sequential Bonferroni significance tests were conducted by means of post hoc testing to investigate variations in coral assemblage composition between depth intervals, while similarity percentage (SIMPER; Clarke 1993) analysis was used to identify the taxa contributing most to inter-reef dissimilarity. Both PERMANOVA and SIMPER analyses were performed in PAST (version 3; Hammer et al. 2001) using Bray–Curtis measures of dissimilarity.

#### Species response curves

The statistical approach described above assumes that changes in coral assemblage composition are independent of age and are a function of water depth. To further investigate the vertical distribution of corals along the water depth gradient, species response curves were produced by generalised additive modelling (GAM) for coral genera for which there were sufficient observations. Models used a cubic smooth spline function with the response variable (relative mass abundance) assigned a quasi-Poisson distribution across all available depth intervals, following recommendations in Lepš and Šmilauer (2003). As the quasi-Poisson distribution uses a canonical log-link function, modelling used untransformed data (Skácelová and Lepš 2014). For each genus, models were compared against the null model at different degrees of complexity (expressed as degrees of freedom; *df* ≤ 6). The optimal model (i.e. the one with the highest parsimony) was selected by comparison of computed Akaike information criterion values (Lepš and Šmilauer 2003). Both the production of species response curves and model selection were performed in CANOCO (version 4.5; Ter Braak and Šmilauer 2002).

#### European settlement

To test the hypothesis that changes in water quality following European settlement (ca. 1850 AD) have altered the composition of coral communities within the PSRC, age–depth models were produced for each core using a flexible Bayesian approach in the software package ‘bacon’ (version 2.2; Blaauw and Christen 2011), run in the R statistical interface (R Development Core Team 2014). Bayesian approaches are increasingly favoured for chronological reconstructions as they consider both age uncertainty and the distributional probability of all available dates (Bennett 1994; Parnell et al. 2011). In ‘bacon’, age–depth models are produced by thousands to millions of Markov chain Monte Carlo iterations, constrained by accumulation rates informed by prior model information, which are then reduced to remove any auto-correlation between model runs (Blaauw and Heegaard 2012). Prior model information was informed by previously published rates of vertical reef accretion within nearshore environments on the GBR (see Perry et al. 2012) and followed recommendations for likely variable rates of accumulation (i.e. low model memory; Blaauw and Christen 2011).

The age–depth models used a total of 96 published radiocarbon dates available for the 21 cores recovered across the PSRC (see Perry et al. 2013; Morgan et al. 2016b; Fig. 2). Model outputs provided age estimates at a 1-cm down-core resolution. Median probability age estimates were used to identify the depth intervals corresponding to European settlement (100 calibrated years before present; cal. yr BP) within each core (Electronic supplementary material; ESM Datasheet S1). Compositional comparisons between assemblages pre- and post-dating European settlement (age) were made by a two-way PERMANOVA, with reef and age as factors. The analysis was performed in PAST (Hammer et al. 2001) using 9999 permutations and Bray–Curtis measures of dissimilarity. To remove any interaction associated with water depth, only depth intervals within which both temporal periods were represented were included in the analysis (ESM Table S1).

## Results

### Sampling evaluation and palaeoecological inventory

**Fig. 3:**
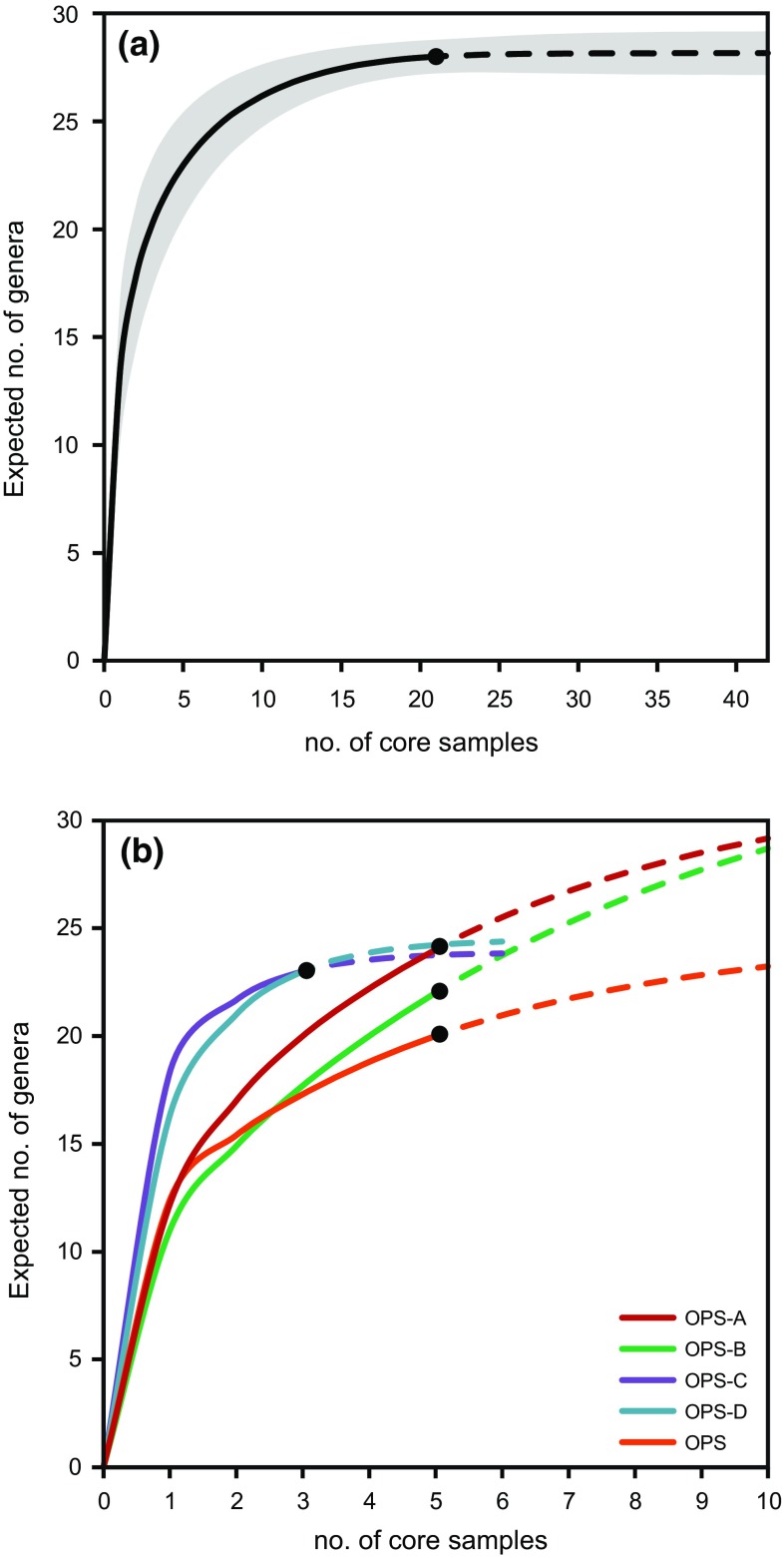
Sample-based rarefaction curves generated for **a** the Paluma Shoals reef complex (PSRC); *shading* represents 95% confidence intervals; and **b** the constituent reefs of the PSRC. Sample reference numbers are indicated by *solid circles*, rarefaction curves by *solid lines*, and extrapolation curves by *dashed lines*

Taxonomic analysis of recovered coral materials identified 59 species of Scleractinia, from 29 genera (ESM Table S2). Sample-based rarefaction confirmed that sampling completeness was achieved across the PSRC and approached saturation for each reef (Fig. 3). The sampling strategy therefore provided a representative record of coral community development and change during vertical reef growth towards sea level across the PSRC.

**Fig. 4:**
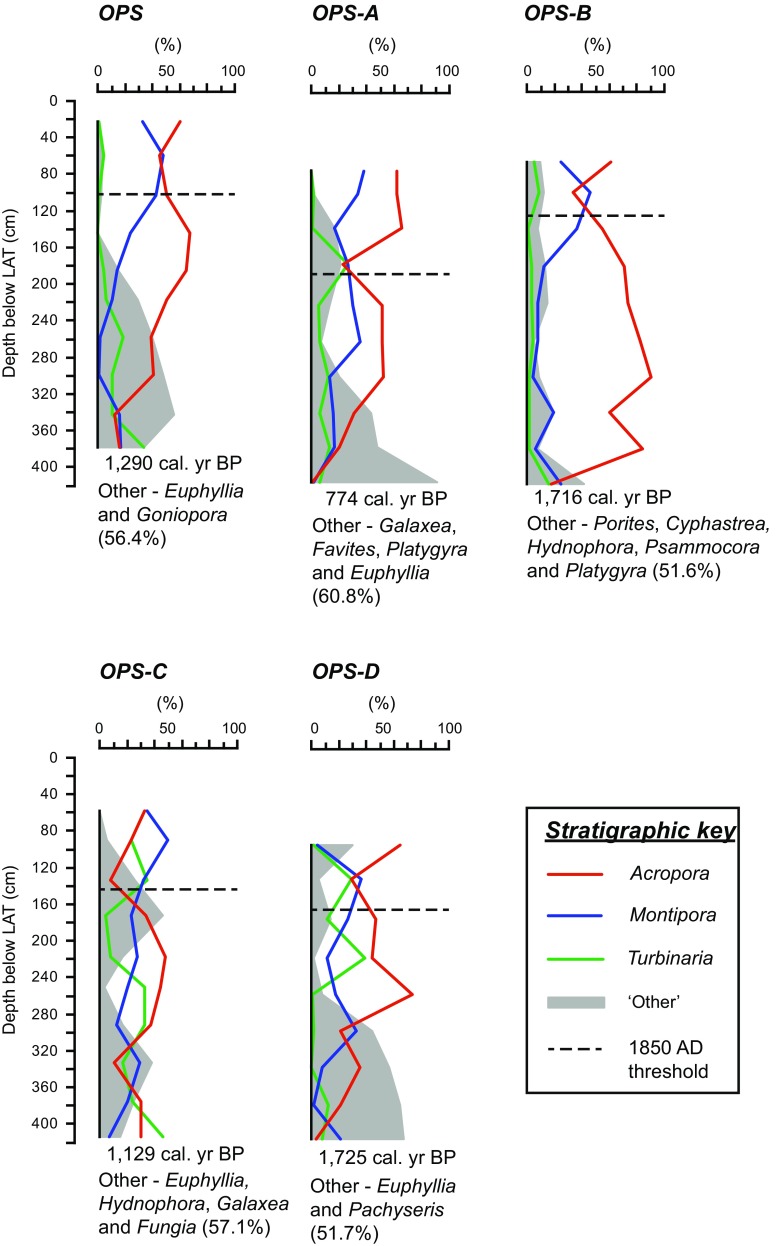
Palaeoecological assemblage records of the five reefs cored within the Paluma Shoals reef complex. The average relative mass abundance of the major coral genera (*Acropora*, *Montipora*, and *Turbinaria*) is presented for each reef. All remaining taxa are grouped as ‘Other’ for presentation purposes. The main taxa (>50%) contributing most to the ‘Other’ group are listed, respectively, in rank order, with their cumulative per cent contribution provided in *brackets*. Average basal radiocarbon ages (calibrated yr before present; cal. yr BP) and elevations of the European settlement 1850 AD threshold (*dashed line*) are presented for each reef. All presented depths have been uncompacted and elevations corrected to lowest astronomical tide (LAT)

The generic richness of the palaeoecological inventories across the PSRC ranged from 20 (OPS) to 24 (OPS-A). The dominant genera (by mass) contributing to the reef framework across the PSRC (Fig. 4; Table 2) were *Acropora* (mainly arborescent species, e.g. *A. muricata* and *A. pulchra*), *Montipora* (mainly platy species, e.g. *M. aequituberculata*), and *Turbinaria* (mainly *T. mesenterina*). Subsidiary, but volumetrically important, contributions were also made by *Cyphastrea serailia*, *Euphyllia* sp., *Favites* spp., *Galaxea fascicularis*, *Goniopora* sp., *Hydnophora microconos*, and *Porites* sp. (Fig. 4; Table 2). Although the palaeoecological assemblages across the PSRC included common taxa (Fig. 4), composition varied significantly among reefs (*p* < 0.001; Table 3a). Inter-reef dissimilarities, identified by SIMPER (Table 4), ranged from 37.7% (between OPS-A and OPS-B) to 51.3% (between OPS and OPS-D). Eight genera were identified as contributing most to the observed inter-reef differences (Table 4). Of these, *Acropora*, *Montipora*, and *Turbinaria* accounted for the majority of the observed inter-reef differences, cumulatively accounting for an average dissimilarity of 41.4%.

**Table 3:** Results of PERMANOVA using coral assemblage data with the factors (a) reef and depth; and (b) age (relative to European settlement) and reef

Source	*df*	SS	MS	Pseudo-*F*	*p*
(a) Two-way PERMANOVA, reef, and depth					
Reef	4	1.631	0.408	3.633	0.0001
Depth	7	1.826	0.261	2.324	0.0001
Interaction	28	1.872	0.067	0.596	0.1911
Residual	126	14.137	0.112		
Total	165	19.465			
(b) Two-way PERMANOVA, pre-/post-European settlement (age), and reef					
Age	1	0.097	0.097	1.286	0.1057
Reef	4	0.741	0.185	2.463	0.0001
Interaction	4	−0.792	−0.198	−2.634	0.0866
Residual	49	3.684	0.075		
Total	58	3.729

**Table 4:** Results of reef-based similarity percentage (SIMPER) analysis

Reef	OPS	OPS-A	OPS-B	OPS-C	OPS-D
OPS	–	**48.1**	**46.8**	**48.8**	**51.3**
OPS-A	*Montipora* (14.8) *Turbinaria* (14.3) *Acropora* (12.6) *Euphyllia* (10.3) *Cyphastrea* (8.5)	–	**37.7**	**39.8**	**45.4**
OPS-B	*Montipora* (15.4) *Acropora* (14.7) *Turbinaria* (14.1) *Euphyllia* (9.7) *Cyphastrea* (9.4)	*Turbinaria* (15.8) *Montipora* (14.2) *Acropora* (10.6) *Cyphastrea* (8.7) *Porites* (8.7)	–	**40.1**	**44.6**
OPS-C	*Turbinaria* (16.9) *Montipora* (14.2) *Acropora* (12.8) *Euphyllia* (10.2) *Cyphastrea* (7.7)	*Turbinaria* (17.1) *Montipora* (11.3) *Acropora* (10.4) *Porites* (8.8) *Euphyllia* (8.4)	*Turbinaria* (19.4) *Acropora* (13.9) *Montipora* (12.9) *Porites* (10.0) *Hydnophora* (7.4)	–	**45.2**
OPS-D	*Turbinaria* (14.2) *Acropora* (14.0) *Montipora* (13.2) *Euphyllia* (12.9) *Galaxea* (9.2)	*Turbinaria* (14.6) *Montipora* (11.6) *Euphyllia* (11.6) *Acropora* (11.4) *Galaxea* (8.9)	*Turbinaria* (14.7) *Acropora* (13.5) *Montipora* (11.7) *Euphyllia* (11.2) *Porites* (8.2)	*Turbinaria* (16.3) *Acropora* (11.7) *Euphyllia* (11.7) *Montipora* (11.3) *Porites* (8.5)	–

Average between-reef dissimilarity (%) is presented in bold with the five coral genera contributing most to the dissimilarity listed below the diagonal. The contribution (%) to the total dissimilarity of each taxon is provided in brackets

### Coral assemblage stratification

There was no significant difference in coral assemblage composition between intervals pre- and post-dating European settlement (*p* = 0.106; Table 3b). Furthermore, tests remained insignificant when performed on both absolute mass and untransformed data. In contrast, coral assemblage composition varied significantly with water depth (*p* < 0.001; Table 3a). There was no significant interaction between reef and depth factors (*p* = 0.191; Table 3a). Pairwise post hoc tests divided coral assemblages into two significantly different groups between 0.8 and 4 m below LAT (ESM Table S3).

**Fig. 5:**
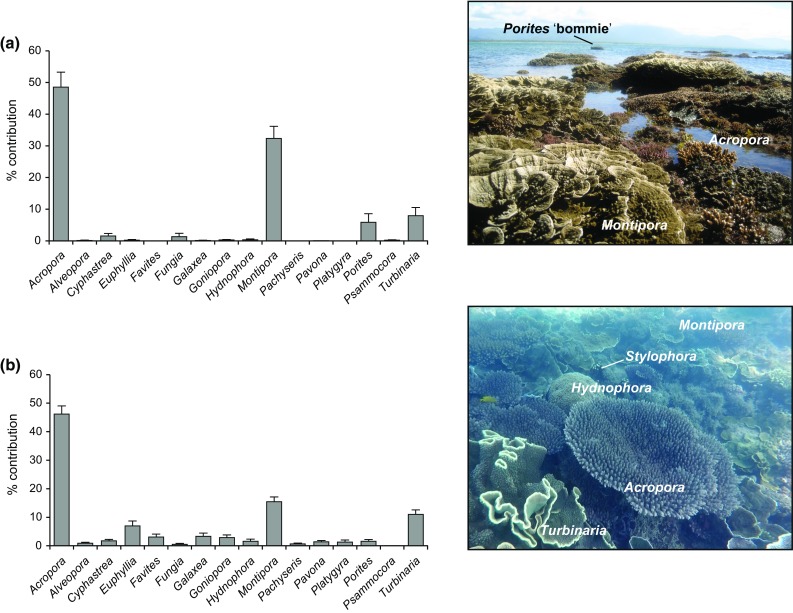
Average relative contribution of coral genera to the overall assemblage composition of the identified: **a** ‘shallow’ assemblage (<1.6 m below lowest astronomical tide; LAT) and **b** ‘deep’ assemblage (>1.6 m below LAT). *Error bars* represent standard error (SE). Representative photographs of analogous modern assemblages within the Paluma Shoals reef complex are presented alongside the corresponding graph

The first group represents a ‘shallow’ assemblage, occurring at depths of less than ~1.6 m below LAT. This assemblage (Fig. 5a) is characterised by high abundances of *Acropora* (48.6 ± 4.7%, mean ± SE) and *Montipora* (32.3 ± 3.8%), with lesser contributions to the overall assemblage composition made by *Turbinaria* (7.9 ± 2.6%) and *Porites* (5.9 ± 2.7%). The second group occurs at depths greater than ~1.6 m below LAT and represents a ‘deep’ assemblage (Fig. 5b). Differences between the identified ‘deep’ and ‘shallow’ assemblages are largely driven by the contribution of ‘other’ coral taxa (excluding *Acropora*, *Montipora*, *Turbinaria*, and *Porites*) to the overall assemblage composition. Collectively, ‘other’ taxa contributed 25.8 ± 2.8% to the composition of the ‘deep’ assemblage, with key contributions to the group made by *Euphyllia*, *Galaxea*, and *Goniopora* (Fig. 5b). Within the ‘shallow’ assemblage, ‘other’ taxa accounted for only 5.3 ± 2.0% of the overall assemblage composition, with *Cyphastrea* and *Fungia* contributing most to the group (Fig. 5a). The abundance of *Montipora* is notably lower within the ‘deep’ assemblage (15.5 ± 1.7%). Contributions by *Acropora* (46.1 ± 2.9%) and *Turbinaria* (11.0 ± 1.6%) to the overall composition of the ‘deep’ assemblage were similar to that of the ‘shallow’ assemblage (Fig. 5).

### Coral responses to water depth

**Fig. 6:**
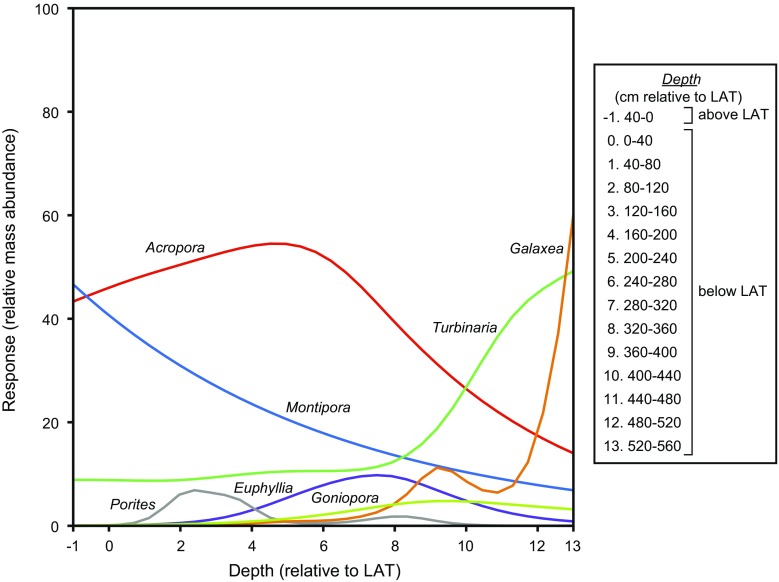
The response (relative mass abundance) of coral genera to water depth (relative to lowest astronomical tide), modelled by generalised additive modelling. Summary statistics are provided in Table 5

Water depth was identified as the key driver of assemblage compositional change and thus the key environmental factor from which to generate response curves. GAMs were fitted to the genera for which there were sufficient observations (Fig. 6). All models were significant at *p* < 0.001 (Table 5) and show coral genera to display four main distributional types along the water depth gradient.

**Table 5:** Summary statistics from generalised additive modelling of the response (relative mass abundance) of coral genera to water depth (relative to lowest astronomical tide)

Response	*Acropora*	*Euphyllia*	*Galaxea*	*Goniopora*	*Montipora*	*Porites*	*Turbinaria*
Selected model	*s(X, 3)	*s(X, 3)	*s(X, 6)	*s(X, 2)	*s(X, 1)	*s(X, 6)	*s(X, 4)
Null model deviance	6120.24	4574.88	3708.05	2442.14	5516.55	2506.98	6282.77
Fitted model deviance	5469.31	3791.17	2260.09	2027.55	4658.37	1972.54	5419.16
*F*	9.78	7.44	10.56	9.13	34.33	4.18	6.12
*p*	<0.001	<0.001	<0.001	<0.001	<0.001	<0.001	<0.001

*s Denotes the selected model (X, *i*), where *i* refers to the degree of complexity (expressed as degrees of freedom). Models with the highest parsimony were selected using the Akaike information criterion

The first type consists of *Acropora* and *Porites*. In general, both genera display a positively skewed uni-modal distribution with optimum abundances occurring at depths of ~1 and 2 m below LAT, respectively. The second distributional type is that of a monotonic decrease in abundance with water depth and is entirely represented by *Montipora*. Abundances of *Montipora* are highest above LAT, where it replaces *Acropora* as the most abundant genus. The third distribution type is represented by *Galaxea* and *Turbinaria* which both exhibit trends of increasing abundance with water depth. This trend is most prominent at depths >3.2 m below LAT. With the exception of depths deeper than ~5 m below LAT, *Turbinaria* is the more abundant of the two genera and is the dominant genus at depths >4 m below LAT. *Turbinaria* also remains a major component of shallower assemblages, but at lower abundances. A negatively skewed uni-modal curve is the final distributional type and is represented by *Euphyllia* and *Goniopora*. In general, these taxa are most abundant at depths deeper than ~3 m below LAT.

## Discussion

### Fossil records as archives of past coral assemblage composition

Fossil coral assemblages are composed of materials originating from life assemblages mixed with those from co-occurring and surrounding death assemblages and are therefore considered to be representative of multiple populations (Edinger et al. 2001; Pandolfi 2002). The reliability with which palaeoecological records can be used to reconstruct the original composition of past coral communities is therefore dependent on the degree to which the fossil assemblage has been mixed over time (Pandolfi and Minchin 1995; Pandolfi 2002). Despite this limitation, a number of studies have shown that fossil coral assemblages provide a good measure of the overall composition of assemblages over timescales relevant to coral reef development and growth, especially where mixing can be inferred to be minimal (Pandolfi 1999; Edinger et al. 2001; Roche et al. 2011).

In this context, the degree to which a fossil assemblage reflects the composition of the original life assemblage is usually highest within low-energy environments (e.g. lagoonal and nearshore settings), where the potential for reworking and transportation is lowest (Scoffin 1992; Pandolfi and Minchin 1995; Edinger et al. 2001). Within the PSRC, limited reworking and transportation of coral materials can be inferred from: (1) the overall preservation of the coral material captured within the recovered cores (ESM Figs. S1–S6), and (2) the persistence of mud-rich terrigenous sediments throughout the entire reef sequence captured in the recovered cores (ESM Video S1). Given these sedimentary and taphonomic signatures, and the effectiveness of the employed sampling strategy as inferred from the sample-based rarefaction analysis (Fig. 3), we are confident that the records presented in this study provide a reliable history of past coral community composition and change within the PSRC.

### Coral assemblages of the Paluma Shoals reef complex

A total of 59 species (ESM Table S2) were identified from the palaeoecological inventory of the PSRC, 95% of which have previously been observed on the inner shelf of the GBR (Sweatman et al. 2007; OBIS 2015). To the best of our knowledge, *Heterocyathus aequicostatus* and *Montipora effusa* have not been previously recorded on the GBR inner shelf, though their distributional ranges are considered to include the zone (OBIS 2015). The richness of genera on the PSRC is higher than previously reported from core records recovered from the landward and more geomorphologically mature reefs of Paluma Shoals (north and south shoal; Perry et al. 2008), but is similar to that reported for other nearshore reefs on the central GBR (e.g. Roche et al. 2011; Ryan et al. 2016). We also note that 90% of the genera identified in this study are present within the contemporary coral communities of the PSRC (Morgan et al. 2016a). Furthermore, the generic richness of the palaeoecological inventory is comparable to the contemporary nearshore coral communities of Middle Reef in Cleveland Bay, approximately 30 km to the south (Browne et al. 2010).

The palaeoecological assemblages of the PSRC are representative of inner-shelf reef communities across the central GBR (DeVantier et al. 2006), which are characterised by mixed assemblages of acroporids (*Montipora,* and locally abundant *Acropora*; Done 1982), dendrophyllids (*Turbinaria*), euphylliids (*Euphyllia* and *Galaxea*), and poritids (*Goniopora* and *Porites*). The composition of inner-shelf coral communities distinguishes them from those associated with mid- and outer-shelf reefs, which are predominantly characterised by assemblages of *Acropora* (Done 1982; DeVantier et al. 2006).

In the PSRC, *Acropora* was one of the most dominant taxa within palaeoecological assemblages (Fig. 4; Table 2). This is consistent with recent surveys of contemporary coral communities that found *Acropora* to account for up to ~30% of the average live coral cover on the PSRC composite reefs (Morgan et al. 2016a). As the analyses in this study were at the genus level, spatial differences and temporal changes in assemblage composition at the species level cannot be explicitly inferred. However, species inventory comparisons suggest that the *Acropora* assemblages of the PSRC (ESM Table S2) are composed of different species (notably arborescent species, e.g. *A. muricata* and *A. pulchra*) from those of the central GBR mid- and outer-shelf reefs, which are characterised by assemblages of *A. hyacinthus/valenciennes* (formerly *A. splendida*) and *A. humilis/Isopora palifera* (formerly *A. palifera*), respectively (Done 1982; DeVantier et al. 2006).

### Vertical trends in nearshore coral distribution

Recent surveys of the contemporary coral communities of the PSRC have shown that coral taxa exhibit clear depth stratification (Morgan et al. 2016a). Specifically, benthic surveys found shallow (<1.5 m below LAT) coral assemblages to be characterised by *Acropora* and *Montipora*, while *Turbinaria* and taxa with sub-massive growth forms (e.g. *Galaxea* and *Goniopora*) were associated with deeper assemblages. These observed trends are remarkably well preserved within the palaeoecological records of the PSRC (Figs. 5, 6). This finding therefore, provides compelling evidence for the argument that nearshore coral communities, and the associated controls of community development, have remained largely unchanged throughout the growth history of the PSRC.

Similar patterns in the vertical distribution of coral taxa have also been reported for contemporary coral communities across the central GBR continental shelf. These patterns have been attributed to intrinsic variations in hydrodynamic energy and, by association, changes in light availability and sedimentation (Yentsch et al. 2002; Wolanski et al. 2005), corresponding to shallowing conditions during vertical reef development towards sea level. Specifically, the palaeoecological record of the PSRC demonstrates the preference of *Euphyllia*, *Galaxea*, *Goniopora*, and *Turbinaria* for deeper environments (approx. >3 m below LAT; Fig. 6). These genera are likely representative of the main coral taxa associated with the early incipient stages of reef development within the PSRC. Available radiocarbon dates (Perry et al. 2013; Morgan et al. 2016b) suggest that reef initiation within the PSRC occurred between ~2000 and 700 cal. yr BP (Fig. 2). Although the precise nature of past regional sea-level dynamics is still debatable, existing sea-level curves suggest that reef initiation within the PSRC reef occurred when palaeowater depths were no greater than 1 m above present-day elevations (i.e. at depths between ~4 and 5 m below present LAT; Lewis et al. 2013, 2015). These reported initiation depths are comparable to recent estimates of maximum photic depths within the coastal zone of the Burdekin region of the GBR, suggesting that benthic light availability is a key environmental control for the initiation of reef development within turbid nearshore environments (Fabricius et al. 2016; Morgan et al. 2016b).

Subsequent shallower stages of vertical reef development within the PSRC are dominated by *Acropora* and *Montipora* (Fig. 6). Due to their ability to settle in dense patches and grow rapidly, these taxa are considered to be opportunistic (Done et al. 2007). Interestingly, the palaeoecological record of the PSRC indicates a decline in the prevalence of *Acropora* as water depth shallows beyond ~2 m below LAT, with *Montipora* becoming the dominant genus following attainment of LAT (Fig. 6). This transition also corresponds with a decline in generic richness between the identified ‘shallow’ and ‘deep’ assemblages (Fig. 5) and is consistent with reported trends of decreasing taxonomic richness with progressive shallowing along the water depth gradient (DeVantier et al. 2006). This shallowing signal is interpreted as a response to increased wave exposure under vertical reef development towards sea level (Done et al. 2007; Roberts et al. 2015; Morgan et al. 2016a) and highlights the importance of considering geomorphological maturity when determining the ecological status of a reef (Perry and Smithers 2011). Under further vertical growth and lateral reef flat development, over time we anticipate that the reef-top communities of the PSRC will transition towards an aerially exposed intertidal coral community dominated by the thermally and high irradiance-tolerant species, *Coelastrea aspera* (formerly *Goniastrea aspera*; Brown et al. 2002; Huang et al. 2014). Such communities are characteristic of contemporary sea-level-constrained nearshore reef flats elsewhere on the central GBR (e.g. Roche et al. 2011; Browne et al. 2010; Palmer et al. 2010).

### Nearshore reef resilience

Several studies have argued that the observed robust nature of nearshore coral communities is a function of the naturally ‘marginal’ environments in which they occur (Larcombe and Woolfe 1999a; Perry et al. 2008, 2009). Under this premise, it is proposed that the naturally high background sedimentary environment occupied by nearshore coral reefs acts to buffer the magnitude of water-quality change to which the corals are exposed (Larcombe and Woolfe 1999a; Orpin and Ridd 2012). Our records found no discernible evidence of generic compositional change within nearshore coral assemblages relative to European settlement. Instead, this study demonstrates the long-term persistence of coral communities and their associated taxa within nearshore environments on the central GBR. These results are consistent with those of other palaeoecological studies derived from both coral (e.g. Perry et al. 2008, 2009; Ryan et al. 2016) and foraminiferal records (e.g. Reymond et al. 2013) from other inner-shelf reefs on the central GBR. Collectively, these studies demonstrate the important role of palaeoecological records for the assessment and contextualisation of both ecological and environmental change on coral reefs.

Furthermore, high rates of sedimentation and chronic low light conditions as a result of light attenuation by suspended sediments can be inferred to have persisted throughout the entire growth history of the PSRC (ESM Video S1). Undoubtedly, these environmental conditions have encouraged and promoted the development of coral communities characterised by taxa which are pre-adapted to conditions of high turbidity within the most nearshore areas of the central GBR (see Browne et al. 2012a and references therein). Indeed, several genera characteristic of the PSRC and other inner-shelf reef communities are known to be able to cope with low light availability and/or high rates of sedimentation (see Anthony and Fabricius 2000; Anthony et al. 2005; Houlbrèque and Ferrier-Pagès 2009; Browne et al. 2012a). Typical adaptive mechanisms include the short-term coping strategies of heterotrophic feeding (e.g. *Coelastrea*), an increased capacity for sediment removal (e.g. *Galaxea*, *Goniopora*, *Porites*, *Platygyra*), and the longer-term strategy of morphological plasticity (e.g. *Turbinaria*). The adaptive potential of certain coral taxa to the sedimentary conditions of the GBR nearshore setting is further demonstrated by the measured growth rates of the common nearshore species *A. muricata* and *M. aequituberculata*, which are comparable to those of their clear-water counterparts (Browne 2012).

### Further considerations

This study supports the premise that water-quality decline following European settlement has not yet impacted the most nearshore reefs of the central GBR (Larcombe and Woolfe 1999a; Orpin and Ridd 2012). The palaeoecological records presented in this study therefore represent important ecological baselines that can be used for monitoring and assessing future ecological change on nearshore coral reefs in the region. Our findings demonstrate the apparently robust nature of nearshore reefs and suggest that their associated coral communities may be more resilient to changes in water quality than those of inshore reefs. To this end, areas where coral communities are likely to be more susceptible to reduced water quality include locations where (1) the local natural sedimentary background conditions are less extreme, such as inshore coral reefs towards the seaward margin of the inshore sediment prism (e.g. Pelorus Island; Roff et al. 2013), and (2) the presence of gyres may potentially restrict flushing times of coastal waters (Clark et al. 2016). Additional palaeoecological coral assemblage records, developed for nearshore coral reefs across the gradient of sediment influence and spectrum of reef geomorphic development, are required to further test the spatial consistencies of the trends reported in this study.

## Electronic supplementary material

Below is the link to the electronic supplementary material.

Supplementary material 1 (EPS 117903 kb)

Supplementary material 2 (EPS 168823 kb)

Supplementary material 3 (EPS 100750 kb)

Supplementary material 4 (EPS 69791 kb)

Supplementary material 5 (EPS 174774 kb)

Supplementary material 6 (EPS 102441 kb)

Supplementary material 7 (MP4 127087 kb)

Supplementary material 8 (XLSX 402 kb)

Supplementary material 9 (DOCX 19 kb)

Supplementary material 10 (DOCX 22 kb)
