# Supplementary material for: Palaeoecological records of coral community development on a turbid, nearshore reef complex: baselines for assessing ecological change
Source: Coral Reefs. 2017 Mar 4;36(3):685–700. doi: 10.1007/s00338-017-1561-1 (PMC6979561; doi:10.1007/s00338-017-1561-1)
Supplement: Supplementary file 9 — Supplementary material 9 (DOCX 19 kb) [file 338_2017_1561_MOESM9_ESM.docx]

Fig. S1 Family: Acroporidae Genus: *Acropora* (a) *A. aculeus* (Dana 1846); (b) *A.austera* (Dana 1846); (c) *A. horrida* (Dana 1846); (d) *A. hyacinthus* (Dana 1846); (e) *A. intermedi*a (Brook 1891); (f) *A. kirstyae* (Veron and Wallace 1984); (g) *A. muricata* (Linnaeus 1758); (h) *A. pulchra* (Brook, 1819); (i) *A. selago* (Studer 1878); (j) *A. solitaryensis* (Veron and Wallace 1984); (k) *A. vaughani* (Wells 1954). Genus: *Alveopora* (l) *Alveopora* sp. (Blainville 1830) Scale bar = 1 cm.

Fig. S2 Family: Acroporidae Genus: *Montipora* (a) *M. aequituberculata* (Bernard 1897); (b) *M. digitata* (Dana 1846); (c) *M. effusa* (Dana 1846); (d) *M. grisea* (Bernard 1897); (e) *M. incrassata* (Dana 1846); (f) *M. informis* (Bernard 1897); (g) *M. mollis* (Barnard 1897); (h) *M. nodosa* (Dana 1846); (i) *M. peltiformis* (Bernard 1897); (j*) M. spongodes* (Bernard 1897); (k) *M. spumosa* (Lamarck 1816); (l) *M. stellata* (Bernard 1897); (m) *M. turgescens* (Bernard 1897). Scale bar = 1 cm.

Fig. S3 Family: Agariciidae Genus: *Leptoseris* (a) *L. explanata* (Yabe and Sugiyama 1941). Genus: *Pavona* (b) *P. cactus* (Forskål 1775); (c) *P. minuta* (Wells 1954). Family: Caryophlliidae, Genus: *Heterocyathus* (d) *H. aequicostatus,* scale bar = 1 mm (Milne Edwards and Haime 1848). Family: Coscinaridae Genus: *Coscinarea* (e) *C. columna* (Dana 1846). Family: Fungiidae, Genus: *Fungia* (f) *Fungia* sp. (Lamarck 1801). Family: Euphylliidae Genus: *Euphyllia* (g) *Euphyllia* sp. (Dana 1846). Genus: *Galaxea* (h) *G. fascicularis* (Linnaeus, 1767). Scale bar = 1 cm, except where indicated.

Fig. S4 Family: Dendrophylliidae Genus: *Balanophyllia* (a) *B.* cf. *bairdiana* (Milne Edwards and Haime 1848). Genus: *Duncanopsammia* (b) *D. axifuga* (Milne Edwards and Haime 1848). Genus: *Turbinaria* (c) *T. bifrons* (Brüggemann 1877); (d) *T. frondens* (Dana 1846); (e) *T. mesenterina* (Lamarck 1816); (f) *T. reniformis* (Bernard 1896). Family: Incertae sedis Genus: *Pachyseris* (g) *P. speciosa* (Dana 1846). Scale bar = 1 cm.

Fig. S5 Family: Merulinidae Genus: *Cyphastrea* (a) *C. serailia* (Forskål 1775). Genus: *Dipsastraea* (b) *D. favus* (Forskål 1775); (c) *D. maritima* (Nemenzo 1971). Genus: *Echinopora* (d) *E. lamellosa* (Esper 1795). Genus: *Favites* (e) *F. pentagona* (Esper 1794); (f) *F. halicora* (Ehrenberg 1834). Genus: *Hydnophora* (g) *H. microconos* (Lamarck 1816); (h) *H. rigida* (Dana 1846). Genus: *Oulophyllia* (i) *Oulophyllia* sp. (Milne Edwards and Haime 1848). Genus: *Platygyra* (j) *P. sinensis* (Milne Edwards and Haime 1848). Family: Psammocoridae Genus: *Psammocora* (k) *P. contigua* (Esper 1797), (l) *P. obtusangula* (Lamarck 1816). Scale bar = 1 cm.

Fig. S6 Family: Lobophylliidae Genus: *Echinophyllia* (a) *E. orpheensis* (Veron and Pichon 1980). Genus: *Lobophyllia* (b) *Lobophyllia* sp. (Bainville 1830). Genus: Oxypora (c) *O. lacera* (Verrill 1864). Family: Poritidae, Genus: *Goniopora* (d) *Goniopora* sp. (Bainville 1830). Genus: *Porites* (e) *Porites* sp. (Link 1807). Family: Pocilloporidae Genus: *Pocillopora* (f) *P. damicornis* (Linnaeus 1758). Genus: *Stylophora* (g) *S. pistillata* (Esper 1797). Scale bar = 1 cm.

**Datasheet S1** Bayesian age-depth modelling results for each core recovered and analysed from the Paluma Shoals reef complex. Models were produced using a total of 96 radiocarbon dates (published in Perry et al. 2013; Morgan et al. 2016), available for the 21 recovered cores. Median probability age estimates were used to determine the interval corresponding to the European settlement, 1850 AD threshold (100 calibrated yr before present).

**Video S1** Video footage showing a typical core collected from the Paluma Shoals reef complex. Coral materials are embedded within a mud-rich sediment matrix, persisting throughout the entire reefal sequence captured by the core. The core penetrated the entire Holocene reef sequence of the reef and terminated in a facies unit of pre-reefal clay.

**References**

Perry CT, Smithers SG, Gulliver P (2013) Rapid vertical accretion on a ‘young’ shore-detached turbid zone reef: Offshore Paluma Shoals, central Great Barrier Reef, Australia. Coral Reefs 32:1143–1148

Morgan KM, Perry CT, Smithers SG, Johnson JA, Gulliver P (2016b) Transitions in coral reef accretion rates linked to intrinsic ecological shifts on turbid-zone nearshore reefs. Geology 44:995–998
