# Supplementary material for: Palaeoecological records of coral community development on a turbid, nearshore reef complex: baselines for assessing ecological change
Source: Coral Reefs. 2017 Mar 4;36(3):685–700. doi: 10.1007/s00338-017-1561-1 (PMC6979561; doi:10.1007/s00338-017-1561-1)
Supplement: Supplementary file 10 — Supplementary material 10 (DOCX 22 kb) [file 338_2017_1561_MOESM10_ESM.docx]

Table S1 Number of core samples per depth interval dating to pre- and post-European settlement (ca. 1850 AD). Rows highlighted in bold indicate the depth interval and sample number used for PERMANOVA analysis (Table 3b)

| **Depth interval (cm below LAT)** | **Pre-European settlement** | **Post-European settlement** |
| --- | --- | --- |
| *0–40* | 0 | 9 |
| ***40–80*** | **5** | **11** |
| ***80–120*** | **10** | **11** |
| ***120–160*** | **17** | **4** |
| *160–200* | 21 | 0 |
| *200–240* | 21 | 0 |
| *240–280* | 21 | 0 |
| *280–320* | 21 | 0 |
| *320–360* | 21 | 0 |
| *360–400* | 19 | 0 |
| *400–440* | 15 | 0 |
| *440–480* | 6 | 0 |
| *480–520* | 2 | 0 |
| *520–540* | 1 | 0 |

Table S2 Palaeoecological inventory of the scleractinian coral taxa of the Paluma Shoals reef complex. Specimen reference plates are available in ESM Figs. S1–S6

| **Acroporidae** | **Agariciidae** | **Lobophylliidae** |
| --- | --- | --- |
| *Acropora aculeus* | *Leptoseris explanata* | *Echinophyllia orpheensis* |
| *Acropora austera* | *Pavona cactus* | *Lobophyllia* sp. |
| *Acropora horrida* | *Pavona minuta* | *Oxypora lacera* |
| *Acropora hyacinthus* | **Caryophylliidae** | **Euphylliidae** |
| *Acropora intermedia* | *Heterocyathus aequicostatus* | *Euphyllia* sp. |
| *Acropora kirstyae* | **Dendrophylliidae** | *Galaxea fascicularis* |
| *Acropora muricata* | *Balanophyllia* cf*. bairdiana* | **Coscinaridae** |
| *Acropora pulchra* | *Duncanopsammia axifuga* | *Coscinaraea columna* |
| *Acropora selago* | *Turbinaria bifrons* | **Pocilloporidae** |
| *Acropora solitaryensis* | *Turbinaria frondens* | *Pocillopora damicornis* |
| *Acropora vaughani* | *Turbinaria mesenterina* | *Stylophora pistillata* |
| *Alveopora* sp*.* | *Turbinaria reniformis* | **Poritidae** |
| *Montipora aequituberculata* | **Merulinidae** | *Goniopora* sp. |
| *Montipora digitata* | *Cyphastrea serailia* | *Porites* sp. |
| *Montipora effusa* | *Dipsastraea favus* | **Psammocoridae** |
| *Montipora grisea* | *Dipsastraea maritima* | *Psammocora contigua* |
| *Montipora incrassata* | *Echinopora lamellosa* | *Psammocora obtusangula* |
| *Montipora informis* | *Favites pentagona* | **Incertae sedis** |
| *Montipora mollis* | *Favites halicora* | *Pachyseris speciosa* |
| *Montipora nodosa* | *Hydnophora microconos* |  |
| *Montipora peltiformis* | *Hydnophora rigida* |  |
| *Montipora spongodes* | *Oulophyllia* sp. |  |
| *Montipora spumosa* | *Platygyra sinensis* |  |
| *Montipora stellata* | **Fungiidae** |  |
| *Montipora turgescens* | *Fungia* sp. |  |

Table S3 Results from post hoc tests using pair-wise PERMANOVAs with sequential Bonferroni significance using coral assemblage data between depth intervals relative to lowest astronomical tide (LAT). Significance values highlighted in bold represent *p* < 0.05

| **Depth category (cm below LAT)** | 80-120 | 120-160 | 160-200 | 200-240 | 240-280 | 280-320 | 320-360 | 360-400 |
| --- | --- | --- | --- | --- | --- | --- | --- | --- |
| 80-120 | — | 0.5953 | 0.0893 | **0.0158** | 0.0502 | **0.0003** | **0.0006** | **0.0007** |
| 120-160 | 0.5953 | — | **0.0234** | **0.0005** | **0.0188** | **0.0001** | **0.0001** | **0.0004** |
| 160-200 | 0.0893 | **0.0234** | — | 0.7715 | 0.719 | 0.4746 | 0.0518 | **0.032** |
| 200-240 | **0.0158** | **0.0005** | 0.7715 | — | 0.7284 | 0.7208 | 0.148 | 0.1799 |
| 240-280 | 0.0502 | **0.0188** | 0.719 | 0.7284 | — | 0.8211 | 0.103 | 0.0858 |
| 280-320 | **0.0003** | **0.0001** | 0.4746 | 0.7208 | 0.8211 | — | 0.4365 | 0.2414 |
| 320-360 | **0.0006** | **0.0001** | 0.0518 | 0.148 | 0.103 | 0.4365 | — | 0.7663 |
| 360-400 | **0.0007** | **0.0004** | **0.032** | 0.1799 | 0.0858 | 0.2414 | 0.7663 | — |
